# Longitudinal trajectories of mental health and loneliness for Australian adolescents with‐or‐without neurodevelopmental disorders: the impact of COVID‐19 school lockdowns

**DOI:** 10.1111/jcpp.13579

**Published:** 2022-02-22

**Authors:** Stephen Houghton, Michael Kyron, David Lawrence, Simon Charles Hunter, John Hattie, Annemaree Carroll, Corinne Zadow, Wai Chen

**Affiliations:** ^1^ 2720 Graduate School of Education The University of Western Australia Perth WA Australia; ^2^ 2720 School of Psychological Sciences The University of Western Australia Perth WA Australia; ^3^ 2720 Centre for Social Impact The University of Western Australia Perth WA Australia; ^4^ 2720 Department of Psychology Glasgow Caledonian University Glasgow UK; ^5^ Graduate School of Education The University of Melbourne Melbourne Vic. Australia; ^6^ School of Education Faculty of Humanities and Social Sciences The University of Queensland Brisbane Qld Australia; ^7^ Mental Health Service Fiona Stanley Hospital Perth WA Australia; ^8^ 3431 Curtin Medical School Curtin University Perth WA Australia; ^9^ School of Medicine University of Notre Dame Australia Fremantle WA Australia

**Keywords:** Coronavirus, adolescents, neurodevelopmental disorders, mental health, loneliness, longitudinal

## Abstract

**Background:**

The impact of COVID‐19 (SARS‐CoV‐2) pandemic school lockdowns on the mental health problems and feelings of loneliness of adolescents with neurodevelopmental disorders (NDDs) is hypothesized to be greater than that of their non‐NDD peers. This two and a half year longitudinal study compared changes in the mental health and loneliness of Western Australian adolescents pre‐COVID‐19 (November 2018 and April 2019), immediately prior to COVID‐19 school lockdowns (March 2020), and post schools reopening (July/August 2020).

**Methods:**

An age‐and‐gender matched sample of 476 adolescents with‐or‐without NDDs completed online assessments for mental health and loneliness.

**Results:**

Adolescents with NDDs reported elevated levels of adverse mental health across all four waves of data collection. These young people experienced little change in mental health problems and feelings of loneliness over time, and any increase during school lockdowns returned to, or fell below pre‐COVID‐19 levels once schools reopened. In comparison, adolescents without NDDs experienced significant increases from a low baseline in depression symptoms, externalizing symptoms, feelings of isolation, and having a positive attitude to being alone, and evidenced a significant decline in positive mental wellbeing. Quality of friendships were unaffected by COVID‐19 school lockdowns for all adolescents regardless of NDD status. Of the adolescents with NDDs, those with Attention‐Deficit/Hyperactivity Disorder reported a significant increase in positive mental wellbeing following school lockdowns.

**Conclusions:**

Adolescents with NDDs emerged relatively unscathed from COVID‐19 school lockdowns and the short term impacts associated with these were not maintained over time. These findings should be considered in the context of this study’s geographical location and the unpredictability of school lockdowns. Learning to live with school lockdowns into the future may be a critical element for further investigation in the context of interventions.

## Introduction

Increasing evidence suggests the onset of the COVID‐19 (SARS‐CoV‐2) pandemic, along with school closures, stay at home orders, social distancing from peers and teachers, and canceling of extra‐curricular activities (collectively known as ‘lockdowns’ henceforth), had deleterious effects on adolescents’ mental health worldwide (e.g. Asanov, Flores, McKenzie, Mensmann, & Schulte, 2021; Ellis, Dumas, & Forbes, 2020; Ravens‐Sieber et al., 2021; Romm, Park, Hughes, & Gentzler, 2021). In previous disease outbreaks such as SARS and MERS the containment measures imposed led to significant increases in risk of mental illness among adolescents and poorer mental health up to nine years later (Brooks et al., 2020; Loades et al., 2020; Rogers et al., 2020). Furthermore, an unintended consequence of these containment measures was increased loneliness. Similarly, COVID‐19 school lockdowns have generated increased feelings of loneliness and disconnect from friends for many adolescents (Ellis et al., 2020). In Australia, over 60% of adolescents ‘frequently’ felt lonely during the COVID‐19 period (Li et al., 2021). Conversely, in Peru no changes in adolescent’s loneliness were found during lockdowns (Magis‐Weinberg, Gys, Berger, Domoff, & Dahl, 2021).

Adolescence is a particularly high‐risk period for psychopathological symptoms developing into full‐blown mental disorders (see Lee et al., 2014). It is also *the* peak period of high risk for loneliness (Houghton et al., 2014). Some adolescents, such as those with neurodevelopmental disorders (NDDs e.g. Attention‐Deficit/Hyperactivity Disorder [ADHD], Specific Learning Disorders [SLD], Autism Spectrum Disorder [ASD]) in mainstream secondary schools are particularly vulnerable to loneliness and heightened risk of developing mental health problems (Arim et al., 2015). They also experience greater psychological distress and adverse mental health following disasters and unpredictable events (Cavallera, Nasir, & Munir, 2020). Daily routines that promote structure, and facilitate social contact with peers and teachers, may be disrupted by COVID‐19 school lockdowns and as such put adolescents with NDDs at significantly heightened risk of adversity (Breaux et al., 2021; Colizzi et al., 2020; Summers et al., 2021).

To date, the impact of COVID‐19 school lockdowns on adolescents with NDDs is relatively unknown (Berard et al., 2021). Most of the studies that include young people with NDDs have been cross‐sectional, focused on ADHD, collected data ‘during’ the pandemic months, and/or lacked comparison groups. Notwithstanding, findings point to worsening emotional wellbeing and mental health and emotional‐mood states (Melegari et al., 2021; Sciberras et al., 2021; Sibley et al., 2021) during the COVID‐19 period. Significant deteriorations have also been reported during school closures in externalizing, aggressive and challenging behaviors, and emotional health among young people with ADHD, ASD, and SLD (Benassi, Bello, Camia, & Scorza, 2021; Berard et al., 2021; Kawaoka et al., 2021). Longitudinal examinations of the impact of COVID‐19 on adolescents with NDDs are scarce. Breaux et al. (2021) collected data from 238 adolescents aged 15–19 years (118 with ADHD) and found significant increases from pre‐COVID‐19 levels in depression, anxiety, inattention, sluggish cognitive tempo, and oppositional type symptoms among adolescents with or without ADHD. Adverse changes returned to pre‐COVID levels however, once stay at home orders were lifted. Adolescents with ADHD and poor emotion regulation were at greatest risk for sustained elevation in externalizing symptoms. Deteriorations in mental health from prolonged school closures have also been reported in longitudinal studies of neuro‐typical adolescents (Hussong, Midgette, Thomas, Coffman, & Cho, 2021; Magson et al., 2021).

Challenges have been created by COVID‐19 mitigation policies, especially those known collectively as lockdowns (Sonuga‐Barke, 2021). Consequently, genuine concerns have been expressed for the mental health of vulnerable populations such as adolescents with NDDs. Public debate around these concerns must, however, be informed by further research and reliable data (Jefsen, Rohde, Nørremark, & Østergaard, 2021; Koenig et al., 2021). This study presents findings from a larger four‐wave longitudinal study capturing change in the mental health and feelings of loneliness in a matched sample of Australian adolescents with or without NDDs. Our first aim was to examine changes in adolescent’s mental health problems (depression symptoms, internalizing and externalizing symptoms, and positive mental wellbeing) and loneliness over time. Our second aim was to examine whether any impact of NDDs status was predictive of change in mental health and feelings of loneliness. We also sought to examine the impact of COVID‐19 lockdowns on different NDDs. Given, the increased vulnerability of adolescents with neurodevelopmental risk to adverse mental health and loneliness, the impact of COVID‐19 was expected to be greater.

## Methods

### Participants and settings

The total sample for this study comprised 476 adolescents who were part of a larger longitudinal project. Four separate data collections were undertaken with these adolescents as they progressed through their school year levels: two collections occurred pre‐COVID‐19 (T1 November 2018, *N* = 1,524; T2 April/May 2019, *N* = 1,670); T3 occurred as schools went into lockdown due to COVID‐19 restrictions (March 2020, *N* = 940); and T4 occurred ~4 weeks post schools reopening (in July/August 2020, *N* = 1,385). When schools locked down in March 2020 (T3), 46% of the larger project sample had completed the assessments. Of these, *N* = 238 had been diagnosed with a NDD (ADHD *n* = 55, SLD *n* = 119, ASD *n* = 23, unknown NDD *n* = 16, and 25 had 2 or more diagnoses) and had completed the T1 and T2 (pre‐COVID‐19) assessments. Once schools reopened following lifting of lockdown, the T4 data collection commenced. On completion of this, the 238 NDDs were age and sex matched to a non‐NDD peer who had also completed all four time point assessments. Propensity score matching using SAS Version 9.4 was used to perform a 1:1 case‐controlled match (Parsons, 2004), involving logistic regression to create propensity scores, and a macro to create several match‐pair samples.

The participants for this study came from 11 secondary schools (9 government schools and 2 nongovernment schools) in Perth, Western Australia (WA). Initially, 15 secondary schools (5 in each of three regions) within a 50 km radius of the Perth city center (i.e. the greater Perth area) were randomly selected out of the 35 available. These 15 schools were contacted to see if they would participate in the research and 11 agreed to be involved. The 11 schools were located across a range of socioeconomic status areas as indicated by their Index of Community Socio‐Educational Advantage (ICSEA). ICSEA is set at an average of 1,000 (*SD* = 100) with higher ICSEA values indicating higher levels of educational advantage of students who go to the school. ICSEA values ranged from 904 to 1191. The demographic characteristics at the T3 commencement of school closures are outlined in Table 1.

**Table 1:** Sample characteristics

	Non‐NDD	NDD
Total	238	238
Gender		
Male	131	131
Female	107	107
Age		
10	2	2
11	25	25
12	25	25
13	68	68
14	50	50
15	50	50
16	18	18
Diagnosis		
None	238	–
ADHD	–	76
SLD	–	134
ASD	–	36
Other	–	19

Twenty‐five adolescents had more than one diagnosis. ADHD, attention deficit hyperactivity disorder; ASD, autism spectrum disorder; SLD, specific learning disorder.

### Procedures

Approval for the study was obtained from the Human Research Ethics Committee of the administering institution, the State Department of Education, and the principals of participating schools. Informed consent and verbal assent was provided by participants. All participants were provided with a unique numerical identification code immediately prior to each of the four separate administrations, which allowed them to log on to the survey. To ensure the correct code was used it was given to each participant by a teacher responsible for overseeing survey administration at each time point. This unique code ensured all information was confidential and that data could be linked across all four waves for analysis. Participants completed the surveys online during school time on four separate occasions over ~28 months.

Inclusion criteria for the NDDs sample included a formal diagnosis by a pediatrician according to DSM IV‐TR or DSM 5 criteria and enrolment in regular mainstream school classes with level 1 minimal support required to function in day‐to‐day activities. During the online survey, students were asked to self‐report if they had a diagnosis of ADHD, ASD, or SLD. Once the survey was completed, the school psychologist, principal, and/or year coordinator in each school confirmed whether this self‐reported NDD status was supported by school records (though schools were not permitted to report on specific NDD diagnoses). At the same time, all participants were reviewed to check for students who may not have self‐reported a formal diagnosis by a pediatrician. For these participants, the school personnel confirmed a diagnosis of a NDD but did not provide specific diagnostic information (i.e. ADHD, ASD, or SLD) because of Department of Education guidelines.

### Measures

#### The Perth A‐loneness Scale (PALs, Houghton et al., 2014)

Participants completed the PALs, a validated 24‐item self‐report measure of adolescent loneliness. The PALs comprises four correlated factors, each with six items. Factor one measures quality of friendships (e.g. ‘My friends will stand by me in almost any difficulty’). Factor two feelings of isolation (e.g. ‘I feel like I do not have a friend in the world’). Factor three, positive attitudes toward being alone (e.g. ‘I have discovered the benefits of being alone’) and Factor four, negative attitudes toward being alone (e.g. ‘When I am all by myself, I wish I had a friend to be with’). Participants respond using a six‐point Likert scale: 1 = never to 6 = always. Reliability across all T1–T4 time points was Quality of friendships (α = .89–.91), isolation (α = .81–.88), positive attitudes toward being alone (α = .82–.87), and negative attitudes toward being alone (α = .75–.82).

#### Children’s Depression Inventory‐2 (self‐report short version; CDI:SR [S] 2 (Kovacs, 2004)

Depressive symptoms were assessed using the CDI:SR [S] 2, a brief self‐report assessment of cognitive, affective, and behavioral symptoms of depression in 7–17‐year olds. Twelve items, each with three separate sentence response options describe participants’ feelings and ideas over the past 2 weeks. Each item is measured on a 3‐point Likert scale (e.g. 0 = I am sad once in a while, 1 = I am sad many times, 2 = I am sad all the time). One item (item 12 ‘I do not feel alone, I feel alone many times, I feel alone all the time’) was removed prior to calculating scores due to content relating to feelings of loneliness, which may bias associations with the PALs. Reliability across T1–T4 time points was α = .85–.87.

#### The Warwick‐Edinburgh Mental Wellbeing Scale (WEMWBS, Tennant et al., 2007)

The WEMWBS comprises 14 positively worded items (e.g. ‘I’ve been feeling cheerful’), to which participants respond using a 5‐point Likert scale (1 = none of the time to 5 = all of the time). Responses are based on participants’ feelings over the previous 2 weeks. One item (‘I've been feeling close to other people’) was removed prior to calculation of total scores due to content relating to feelings of loneliness. Reliability across T1–T4 time points was α = .85–.89.

#### The Strengths and Difficulties Questionnaire (SDQ, Goodman, 1997)

This measured externalising and internalizing symptoms. To measure externalizing symptoms, items which captured conduct problems and hyperactivity were summed. To measure internalizing symptoms, emotional problems and peer relationship problems were summed. Adolescents self‐reported symptoms on a three‐point Likert scale (0 = almost never, 1 = sometimes, 2 = often), with more significant externalizing and internalizing symptoms indicated by higher scores. Reliability across T1–T4 time points was externalizing (α = .77–.81) and internalizing (α = .76–.79).

### Analyses

Linear mixed models were fit to the data to assess differences in change over time for mental health and loneliness in adolescents with or without NDDs. Change over time was determined with reference to pre‐COVID‐19 at Time 2 (April/May 2019), as this was the closest assessment in time to the beginning of COVID‐19. Further, reporting of changes over time has focused on Time 2 to Time 4. Separate growth models were created for each variable, with random intercepts and slopes included to account for individual variability. Models were first run to examine the magnitude of change over time for adolescents with or without NDDs separately. Further, follow‐up models were fit to the data to assess whether the magnitude of change over time was significantly different between NDD and non‐NDD groups, through a difference‐in‐differences (DID) approach (Warton, Parker, & Karter, 2016) that allows for examination of the extent to which change over time in an outcome can be attributed to group membership. In these models, change over time in symptoms were interpreted with reference to symptoms at Pre‐COVID for adolescents without NDDs. Follow‐up mixed models were fit to the data to examine differences in change over time for each NDD diagnosis compared with adolescents without NDDs. Adolescents with more than one diagnosis were excluded from this analysis (*n* = 25) to examine the individual changes over time for each diagnosis. Effect sizes are calculated based on within‐subjects pooled standard deviation and changes in means at schools closed and schools reopened compared with Pre‐COVID‐19 levels. The magnitude of effects interpreted are based on Cohen’s guidelines (i.e. weak = 0.2, medium = 0.5, strong = 0.8). Individual mixed models assessing change over time individually for each diagnosis is reported in the Supporting Information. To account for multiple tests assessing change over time for multiple outcomes and the increased likelihood of detecting statistically significant effects, a Benjamini–Hochberg approach was used to incrementally adjust *p*‐value thresholds for significance (Benjamini & Hochberg, 1995). Multiple testing calculations have been outlined in the Supporting Information.

Of the 476 adolescents who completed surveys at Pre‐COVID, 240 (50.4%) completed surveys at school lockdowns, and 334 (70.2%) completed surveys at the reopening of schools. Compared to Pre‐COVID levels, adolescents with missing data at lockdowns or reopening did not have statistically different depression symptoms and positive mental wellbeing. However, average age was slightly lower for adolescents who completed surveys at school lockdowns *t*(474) = 2.18, *p* = .03. Chi‐square tests of differences found no significant differences in survey completion by gender. Missing data were handled with maximum likelihood, which has been shown to produce largely equivalent results when compared with multiple imputation (Newman, 2003). Correlations between all measures are reported in Table S1 at Time 2 due to the highest number of respondents. Descriptive statistics have been reported at all‐time points in Table S2. All analyses were conducted using SAS Version 9.4.

## Results

### Changes in mental health over time based on NDD status

**Figure 1:**
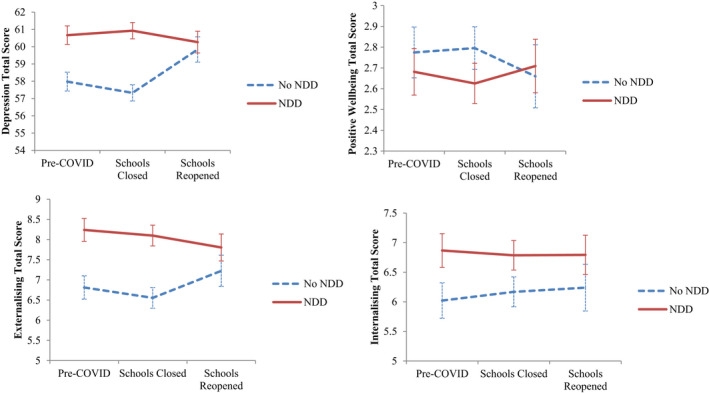
Mean trajectories of depression symptoms, positive mental wellbeing, internalizing and externalizing symptoms prior to and post school reopening for adolescents with and without NDDs

In general, adolescents with or without NDDs responded to school lockdowns differently. Lockdowns increased morbidities in adolescents without NDDs (these had low baseline symptoms), but did not worsen for adolescents with NDDs (already had elevated baseline symptoms). As shown in Figure 1, the trend was of increasing depression symptoms for adolescents without NDDs, but comparatively stable levels of high depression symptoms for adolescents with NDDs. Further, there was a trend of decreasing positive mental wellbeing for adolescents without NDDs post schools reopening, but increasing positive mental wellbeing for adolescents with NDDs. Generally, the trends for adolescents with NDDs suggest a return to, or below, pre‐COVID‐19 levels for mental health post schools reopening.

Tests for changes over time for adolescents with or without NDDs individually have been reported in Table 2. At post schools reopening, adolescents without NDDs reported significant increases in depression symptoms (*B* = 2.12, *p* = .009) and externalizing symptoms (*B* = 0.71, *p* = .001), but not internalizing symptoms (*B* = 0.25, *p* = .236). In addition, they reported significant decreases in positive mental wellbeing (*B* = −0.12, *p* = .005). No significant changes over time were evident for the NDD group. Effect sizes for changes over time were small for both groups.

**Table 2:** Unstandardized estimates of change in mental health over time for adolescents with or without NDDs

	Depression symptoms						Positive mental wellbeing					
	Non‐NDD			NDD			Non‐NDD			NDD		
	Estimate	*p*	*d*	Estimate	*p*	*d*	Estimate	*p*	*d*	Estimate	*p*	*d*
*Time*												
Pre‐COVID	(ref)	–	–	(ref)	–	–	(ref)	–	–	(ref)	–	–
Schools closed	0.93	.342	.05	0.59	.506	.02	–0.06	.246	.03	–0.07	.116	.10
Schools reopened	**2.12**	.**009**	.**13**	0.23	.778	.03	–**0.12**	.**005**	.**18**	0.03	.523	.05

	Externalizing symptoms						Internalizing symptoms					
	Non‐NDD			NDD			Non‐NDD			NDD		
	Estimate	*p*	*d*	Estimate	*p*	*d*	Estimate	*p*	*d*	Estimate	*p*	*d*
*Time*												
Pre‐COVID	(ref)	–	–	(ref)	–	–	(ref)	–	–	(ref)	–	–
Schools closed	0.19	.472	.06	−0.03	.922	.04	0.31	.232	.04	0.10	.708	.02
Schools reopened	**0.71**	.**001**	.**10**	−0.22	.317	.11	0.25	.236	.06	0.02	.950	.02

*d* = Cohen’s *d* comparing magnitude of change at schools closed and schools reopened compared to pre‐COVID. Boldfaced *p*‐values indicate statistically significant associations when accounting for multiple testing.

### Changes in loneliness over time based on NDD status

**Figure 2:**
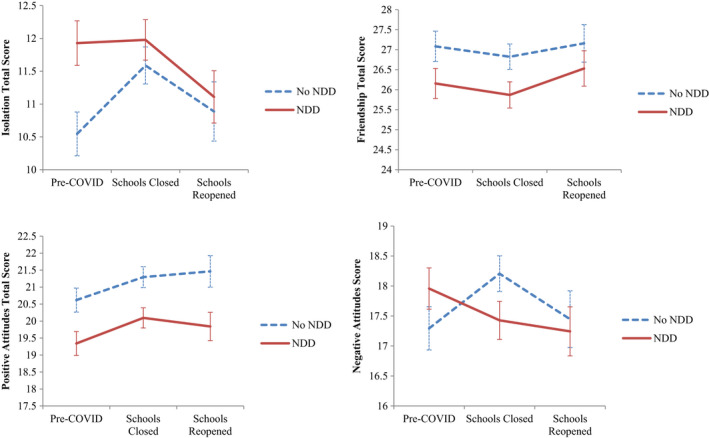
Mean trajectories of loneliness before and after COVID‐19 for NDD and non‐NDD adolescents

The trends for each loneliness dimension show a notable spike in feelings of isolation, and both negative and positive attitudes toward being alone at the commencement of school lockdowns for adolescents without NDDs (see Figure 2). A decrease in feelings of isolation was evident at post schools reopening, along with an increase in quality of friendships for both adolescents with or without NDDs. Trends for adolescents with NDDs suggest that feelings of isolation and a negative attitude to being alone were below pre‐COVID‐19 levels, while quality of friendships and a positive attitude toward being alone were above pre‐COVID‐19 levels.

Tests for changes over time (Table 3) for adolescents with or without NDDs individually identified no significant differences in quality of friendships, and positive and negative attitudes toward being alone. For adolescents without NDDs isolation increased significantly at school lockdowns (*B* = 1.15, *p* = .005) and post schools reopening (*B* = 0.38, *p* = .257) compared to pre‐COVID levels, and positive attitudes increased at post schools reopening (*B* = .901, *p* = .019). For adolescents with NDDs, there were no significant changes over time in each aspect of loneliness.

**Table 3:** Unstandardized estimates of change in loneliness over time for adolescents with or without NDDs

	Isolation						Positive attitudes					
	Non‐NDD			NDD			Non‐NDD			NDD		
	Estimate	*p*	*d*	Estimate	*p*	*d*	Estimate	*p*	*d*	Estimate	*p*	*d*
*Time*												
Pre‐COVID	(ref)	–	–	(ref)	–		(ref)	–		(ref)	–	
Schools Closed	**1.15**	.**005**	.**21**	0.09	.830	.01	0.325	.487	.12	0.53	.190	.12
Schools Reopened	0.38	.257	.07	−0.60	.133	.14	**0.901**	.**019**	.15	0.47	.205	.15

	Quality Friendships						Negative Attitudes					
	Non‐NDD			NDD			Non‐NDD			NDD		
	Estimate	*p*	*d*	Estimate	*p*	*d*	Estimate	*p*	*d*	Estimate	*p*	*d*
*Time*												
Pre‐COVID	(ref)	–		(ref)	–		(ref)	–		(ref)	–	
Schools Closed	0.004	.993	.04	0.004	.993	.04	0.585	.179	.16	−0.46	.311	.09
Schools Reopened	0.02	.966	.01	0.32	.471	.**06**	0.105	.768	.03	−0.70	.101	.12

*d* = Cohen’s *d* comparing magnitude of change at schools closed and schools reopened compared to pre‐COVID. Boldfaced *p*‐values indicate statistically significant associations when accounting for multiple testing.

### Tests of changes over time in mental health based on NDD status

DID tests examined whether the magnitude of change over time in symptoms were significantly different between adolescents with or without NDDs (Table 4). The magnitude of change over time between groups from Pre‐COVID to school lockdowns in depression, internalizing, and externalizing symptoms was nonsignificant. From Pre‐COVID to post schools reopening, adolescents with NDDs reported significantly different change in positive mental wellbeing compared to adolescents without NDDs (*B* = 0.15, *p* = .012), with non‐NDDs experiencing a negative change over time and NDDs experiencing a marginal positive shift on average when schools reopened. At Pre‐COVID, adolescents with NDDs reported higher levels of depression (*B* = 2.69, *p* = .037), internalizing (*B* = 1.41, *p* < .001), and externalizing symptoms (*B* = 0.85, *p* = .021).

**Table 4:** Mixed models assessing change over time in loneliness for the full sample and adolescents with NDDs

	Depression		Mental Wellbeing		Externalising Symptoms		Internalizing Symptoms	
	Estimate	*p*	Estimate	*p*	Estimate	*p*	Estimate	*p*
NDD*Time								
Pre‐COVID	**2.69**	.**037**	−0.09	.103	**1.39**	**<.001**	**0.85**	.**018**
Schools Closed	−0.30	.830	−0.01	.866	−0.09	.796	−0.19	.609
Schools Reopened	1.95	.106	**0.15**	.**012**	−**0.90**	.**005**	−0.23	.487

	Quality friendship		Isolation		Positive attitude		Negative attitude	
	Estimate	*p*	Estimate	*p*	Estimate	*p*	Estimate	*p*
NDD*Time								
Pre‐COVID	−.929	.124	**1.38**	.**005**	−**1.28**	.**017**	0.66	.209
Schools closed	0.09	.902	−1.01	.106	0.18	.786	−1.04	.123
Schools reopened	0.41	.523	−1.06	.053	−0.56	.336	−0.76	.200

Non‐NDD adolescents at Pre‐COVID are reference category (Non‐NDD = 0, NDD = 1). Boldfaced *p*‐values indicate statistically significant associations when accounting for multiple testing.

### Tests of changes over time in loneliness based on NDD status

Tests for changes over time (Table 4) identified nonsignificant differences in the magnitude of change between groups in terms of quality of friendships, isolation, and positive and negative attitudes toward being alone, from Pre‐COVID to school lockdowns and schools reopening. At Pre‐COVID, adolescents with NDDs reported significantly higher levels of isolation (*B* = 1.38, *p* = .005) and lower positive attitudes toward being alone (*B* = −1.28, *p* = .017).

### Changes for mental health based on individual diagnoses

**Figure 3:**
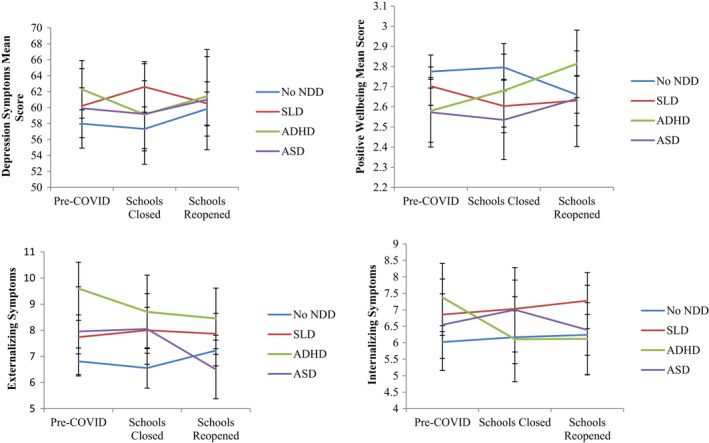
Mean change over time based on NDD diagnosis. ADHD, attention deficit hyperactive disorder; ASD, autism spectrum disorder; SLD, specific learning disorder

Mean changes over time according to NDD diagnosis is shown in Figure 3. Tests for changes over time (Table S3) identified no statistically significant differences in depression, internalizing and externalizing symptoms at school closures for each NDD diagnostic category compared to adolescents without NDDs from Pre‐COVID to school lockdowns and post schools reopening. However, adolescents with ADHD reported a significantly higher shift in positive mental wellbeing from Pre‐COVID to post schools reopening (*B* = 0.35, *p* = .015) compared to adolescents without NDDs. Statistical significance should be interpreted with caution, as this estimate became statistically nonsignificant when accounting for multiple testing.

At Pre‐COVID, adolescents with ADHD reported significantly lower levels of positive mental wellbeing (*B* = −0.22, *p* = .018), higher depression symptoms (*B* = 5.17, *p* = .013), internalizing symptoms (*B* = 1.63, *p* = .005), and externalizing symptoms (*B* = 3.015, *p* < .001) compared to adolescents without NDDs. Further, adolescents with a SLD reported significantly higher internalizing symptoms (*B* = 0.83, *p* = .048) and externalizing symptoms (*B* = 0.92, *p* = .030) compared to adolescents without NDDs at Pre‐COVID. No significant differences were evident for adolescents with ASD.

Lastly, individual growth models were fit for each diagnosis to assess change over time (Table S4). Adolescents with ADHD reported increases in positive mental wellbeing, Pre‐COVID to post school reopening (*B* = 0.23, *p* = .003), and decreases in externalizing symptoms (*B* = 1.15, *p* = .019) (see Table S4). No significant changes over time were evident for adolescents with SLD or ASD.

### Changes for loneliness based on individual diagnoses

**Figure 4:**
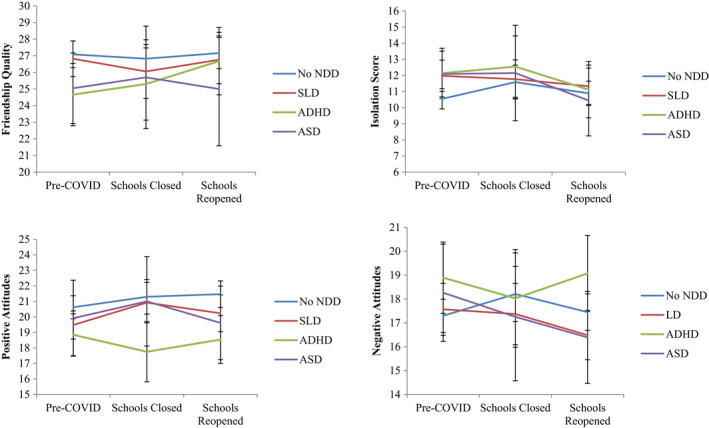
Mean change in loneliness over time based on NDD diagnosis. ADHD, attention deficit hyperactive disorder; ASD, autism spectrum disorder; SLD, specific learning disorder

Mean changes in symptoms over time have been reported in Figure 4 based on NDD diagnosis. There were no significant differences in the magnitude of change in aspects of loneliness for specific NDD diagnoses over time, compared to adolescents without NDDs (Table S5). At Pre‐COVID, adolescents with ADHD reported lower friendship quality (*B* = −3.03, *p* = .002) and positive attitudes to being alone (*B* = −1.76, *p* = .042), and higher isolation (*B* = 2.14, *p* = .009) and negative attitudes (*B* = 1.90, *p* = .025) compared to adolescents without NDDs. Further, adolescents with a SLD reported significantly higher Pre‐COVID isolation (*B* = 1.50, *p* = .015) compared to adolescents without NDDs. No significant differences were evident for adolescents with ASD.

## Discussion

It has been argued that the unpredictability of COVID‐19 school lockdowns may put adolescents with NDDs at greater risk for adverse mental health (Breaux et al., 2021; Melegari et al., 2021). However, adolescents with NDDs in this study did not report significant changes in their mental health over time because of school lockdowns. Rather, adolescents without NDDs experienced a significant increase in depressive symptoms from school lockdowns to post schools reopening. Pre‐COVID‐19 depression symptoms were already high among adolescents with NDDs prior to school lockdowns and remained so across all waves of data collection. School lockdowns did not excessively exacerbate their problems. This reflects the generally higher rates of depression among adolescents with ADHD, ASD, and SLD (Hosozawa, Sacker, & Cable, 2021; Jerrell, McIntyre, & Park, 2015; Visser et al., 2020) and highlights that they continue to be a population at risk. Furthermore, adolescents with NDDs evidenced no change in externalizing symptoms and positive mental wellbeing over time, which contrasts with other studies involving adolescents with ADHD or ASD during COVID‐19 restrictions (e.g. Berard et al., 2021; Kawaoka et al., 2021). This may be due to the heterogeneity known to exist within and between NDDs as a collective category, or that lockdowns were fewer and shorter and without major catastrophic health and social crises in WA compared to other parts of the world. Similar to Breaux et al. (2021), however, mental health difficulties for adolescents with NDDs returned to, or below, pre‐COVID‐19 levels post schools reopening.

School lockdowns may increase risk of poor mental health via increasing loneliness as a result of a reduction in the opportunities for adolescents to interact with peers, and to integrate into the peer group (Orben, Tomova, & Blakemore, 2020). Feeling connected to friends was important for adolescents during COVID‐19 (Ellis et al., 2020; Magson et al., 2021) and in our study, adolescents without NDDs reported significant increases in feelings of isolation as schools locked down. Conversely, adolescents with NDDs did not evidence such increases, possibly because lock downs provided respite from the difficult, stressful, and often threatening face‐to‐face peer interactions they encounter on a daily basis (Foulkes & Blakemore, 2021).

Research shows the peer interaction difficulties experienced by adolescents with NDDs limits the number and quality of their friendships and leads to heightened feelings of loneliness generally (Capodieci et al., 2019; Elmose & Lasgaard, 2017; Lasgaard et al., 2010). As our study revealed, adolescents with NDDs had higher levels of loneliness pre COVID lockdown and this may be why they were not as affected as adolescents without NDDs who suddenly found themselves isolated at lockdown. In addition, quality of friendships (i.e. having reliable, trustworthy, supportive friends) did not change over time for adolescents with NDDs, which may reflect the limited number and quality of friendships they have. Generally, social media use to keep in touch with peers increased during COVID‐19 lockdowns (Cauberghe, Van Wesenbeeck, De Jans, Hudders, & Ponnet, 2021) and adolescents with NDDs use social media significantly more than their neuro‐typical peers (Nereim, Bickham, & Rich, 2019). Connecting virtually with friends, irrespective of number, may not only have provided a means to maintain quality friendships, it may also have helped adolescents with NDDs to feel more connected to others because everyone was staying home and in doing so found social interactions difficult.

The picture emerging from our study is that overall, adolescents with NDDs in WA were relatively unaffected by COVID‐19 school lockdowns. This may be because of initially higher levels of adverse mental health and loneliness compared to adolescents without NDDs; levels, which remained high over time. However, adolescents with ADHD appeared to benefit from school lockdowns, which is in contrast to the findings of Breaux et al. (2021). When schools reopened in our study, adolescents with ADHD reported significantly higher positive mental wellbeing. Spending more time at home during lockdown may have increased household tensions and problematic child/parent relationships, both of which are known to contribute to lower levels of mental wellbeing (Behrmann et al., 2021). Raising a child with ADHD is challenging and associated with higher parenting stress compared with raising a child with another disorder/illness or a healthy child (see Perez Algorta et al., 2018). Hence, returning to more predictable structured school routines when schools reopened meant less disruptions, parental involvement, and reliance on self‐regulated learning (Sciberras et al., 2021; Sibley et al., 2021) and this may be why adolescents with ADHD in our study reported increases in positive mental wellbeing.

A major strength of this study is that it addresses the absence of longitudinal studies examining the impact of COVID‐19 on adolescents with NDDs. Nevertheless, there are limitations that must be acknowledged. First, full diagnostic information was not available for all of our sample. Furthermore, 25 young people had more than one diagnosed disorder, giving rise to issues of comorbidity. Second, the ceiling effect of our measures may have limited detection of deteriorations in those with already elevated baseline scores; at the same time, deteriorations expressed in symptoms or impairments may not have been captured by the range of our measures. Third, our data are based on adolescents self‐report and may be subject to bias and poor recall. However, internalizing experiences require insight into the subjective dispositions that can be difficult to obtain from third parties such as parents and teachers who have great difficulty perceiving the internal world of their children (Baldwin & Dadds, 2007). Fourth, this study sought to provide comprehensive assessment of a wide range of mental health outcomes. Repeated analyses within a single sample however may introduce multiple testing problems that increase chances of Type 1 errors. When applying multiple testing corrections, only a single estimate originally deemed statistically significant was flagged. Specifically, change over time in positive mental wellbeing for adolescents with ADHD was found to be statistically significant compared to adolescents without an NDD when an alpha of 0.05 was used, but not when more stringent criteria were applied. However, in follow‐up tests assessing change over time for each diagnosis individually, positive mental wellbeing was found to significantly increase for adolescents with ADHD even when accounting for multiple testing. Future research should examine whether these findings are replicated in different samples. Finally, rates of COVID‐19 infections and deaths were relatively low in Australia compared to other countries and our results may not be widely generalizable.

Taken together, our findings show that COVID‐19 school lockdowns did not adversely and excessively impact adolescents with NDDs in WA and any increases in adverse mental health and loneliness that did occur, were not maintained over time. Moreover, adolescents with ADHD appeared to benefit from school lockdowns in terms of their positive mental wellbeing. Although unpredictable, school lockdowns in WA were relatively short (9 weeks). Nevertheless, this was longer than Breaux et al.'s (2021) approximate 6 week maximum. Future research should compare the impact of different lengths of COVID‐19 school lockdowns, especially with more severely hit areas than in our study, such as New South Wales and Victoria. Future research should also recruit larger samples of adolescents with NDDs in order to more closely examine how changes may differ among adolescents with differing NDD diagnoses.

## Supporting information


**Table S1.** Correlations and descriptive statistics for measures at Pre‐COVID.
**Table S2.** Mean (*SD*) for both NDD and non‐NDD groups.
**Table S3.** Comparisons of changes over time in mental health dependent on NDD diagnosis.
**Table S4.** Mixed models for assessing change over time for each diagnosis.
**Table S5.** Comparisons of changes over time in loneliness dependent on NDD diagnosis.
**Appendix S1.** *p* Value calculation for Tables 2 and 3.
**Appendix S2.** *p* Value calculation for Table 4.
**Appendix S3.** *p* Value calculation for Table S3.
**Appendix S4.** *p* Value calculation for Table S4.
**Appendix S5.** *p* Value calculation for Table S5.
