# Supplementary material for: Longitudinal trajectories of mental health and loneliness for Australian adolescents with‐or‐without neurodevelopmental disorders: the impact of COVID‐19 school lockdowns
Source: J Child Psychol Psychiatry. 2022 Feb 22;63(11):1332–43. doi: 10.1111/jcpp.13579 (PMC9790479; doi:10.1111/jcpp.13579)
Supplement: Supplementary file 1 — Table S1. Correlations and descriptive statistics for measures at Pre‐COVID. Table S2. Mean (SD) for both NDD and non‐NDD groups. Table S3. Comparisons of changes over time in mental health dependent on NDD diagnosis. Table S4. Mixed models for assessing change over time for each diagnosis. Table S5. Comparisons of changes over time in loneliness dependent on NDD diagnosis. Appendix S1. p Value calculation for Tables 2 and 3. Appendix S2. p Value calculation for Table 4. Appendix S3. p Value calculation for Table S3. Appendix S4. p Value calculation for Table S4. Appendix S5. p Value calculation for Table S5. [file JCPP-63-1332-s001.docx]

**Supporting Information**

**Adjusted *p* values for mixed models**

**Appendix S1.** ***P* value calculation for Table 2 and Table 3**

| P-Values | Rank | Adjusted Threshold |
| --- | --- | --- |
|  |  |  |
| 0.001 | 1 | 0.01087 |
| 0.005 | 2 | 0.021739 |
| 0.005 | 3 | 0.032609 |
| 0.009 | 4 | 0.043478 |
| 0.019 | 5 | .05 |
| 0.101 | 6 |  |
| 0.116 | 7 |  |
| 0.133 | 8 |  |
| 0.179 | 9 |  |
| 0.19 | 10 |  |
| 0.205 | 11 |  |
| 0.232 | 12 |  |
| 0.236 | 13 |  |
| 0.246 | 14 |  |
| 0.257 | 15 |  |
| 0.311 | 16 |  |
| 0.317 | 17 |  |
| 0.342 | 18 |  |
| 0.471 | 19 |  |
| 0.472 | 20 |  |
| 0.487 | 21 |  |
| 0.506 | 22 |  |
| 0.523 | 23 |  |
| 0.708 | 24 |  |
| 0.768 | 25 |  |
| 0.778 | 26 |  |
| 0.83 | 27 |  |
| 0.922 | 28 |  |
| 0.95 | 29 |  |
| 0.966 | 30 |  |
| 0.993 | 31 |  |
| 0.993 | 32 |  |

| **Appendix S2.** **P value calculation for Table 4** | | | | |
| --- | --- | --- | --- | --- |
| **P-Values** | **Rank** | **Adjusted Threshold** | |  |
| < .001 | 1 |  | 0.010417 |  |
| 0.003 | 2 |  | 0.020833 |  |
| 0.005 | 3 |  | 0.03125 |  |
| 0.012 | 4 |  | 0.041667 |  |
| 0.017 | 5 |  | 0.05 |  |
| 0.021 | 6 |  | 0.05 |  |
| 0.037 | 7 |  | 0.05 |  |
| 0.053 | 8 |  |  |  |
| 0.103 | 9 |  |  |  |
| 0.106 | 10 |  |  |  |
| 0.106 | 11 |  |  |  |
| 0.123 | 12 |  |  |  |
| 0.124 | 13 |  |  |  |
| 0.2 | 14 |  |  |  |
| 0.209 | 15 |  |  |  |
| 0.336 | 16 |  |  |  |
| 0.505 | 17 |  |  |  |
| 0.523 | 18 |  |  |  |
| 0.546 | 19 |  |  |  |
| 0.665 | 20 |  |  |  |
| 0.786 | 21 |  |  |  |
| 0.83 | 22 |  |  |  |
| 0.866 | 23 |  |  |  |
| 0.902 | 24 |  |  |  |

**Table S1**

*Correlations and descriptive statistics for measures at Pre-COVID.*

|  | 1. | 2. | 3. | 4. | 5. | 6. | 7. | 8. |
| --- | --- | --- | --- | --- | --- | --- | --- | --- |
| 1. Depression | 1 | - | - | - | - | - | - | - |
| 1. Wellbeing | -0.67*** | 1.00 | - | - | - | - | - | - |
| 1. Externalising | 0.62*** | -0.53*** | 1.00 | - | - | - | - | - |
| 1. Internalising | 0.70*** | -0.61*** | 0.55*** | 1.00 | - | - | - | - |
| 1. Friendships | 0.57*** | -0.54*** | 0.38*** | 0.60*** | 1.00 | - | - | - |
| 1. Isolation | -0.53*** | 0.56*** | -0.32*** | -0.53*** | -0.67*** | 1.00 | - | - |
| 1. Positive | 0.03 | 0.10* | -0.03 | 0.10* | 0.06 | -0.02 | 1.00 | - |
| 1. Negative | 0.38*** | -0.29*** | 0.32*** | 0.35*** | 0.41*** | -0.23*** | -0.25*** | 1.00 |

Note. * *p* < .05; *** *p* < .001..

**Table S2**

*Mean (SD) for both NDD and non-NDD groups.*

|  | Time 1 | | Time 2 | | Time 3 | | Time 4 | |
| --- | --- | --- | --- | --- | --- | --- | --- | --- |
|  | NDD | Non-NDD | NDD | Non-NDD | NDD | Non-NDD | NDD | Non-NDD |
| 1. Depression | 56.63 (13.5) | 61.41 (14.1) | 57.98 (13.8) | 60.67 (13.9) | 57.33 (14.1) | 60.93 (13.7) | 59.84 (14.0) | 60.26 (13.9) |
| 1. Wellbeing | 2.80 (0.6) | 2.69 (0.6) | 2.77 (0.6) | 2.68 (0.6) | 2.80 (0.6) | 2.63 (0.6) | 2.66 (0.6) | 2.71 (0.6) |
| 1. Externalising | 8.08 (3.93) | 6.74 (3.72) | 8.24 (3.98) | 6.81 (4.00) | 8.10 (3.84) | 6.55 (3.95) | 7.80 (3.60) | 7.22 (3.84) |
| 1. Internalising | 6.79 (3.19) | 5.73 (3.91) | 6.87 (3.79) | 6.02 (3.91) | 6.79 (3.76) | 6.17 (4.11) | 6.79 (3.76) | 6.24 (4.02) |
| 1. Friendships | 27.34 (6.5) | 26.17 (7.0) | 27.08 (6.3) | 26.16 (6.6) | 26.82 (5.9) | 25.87 (7.0) | 27.16 (6.2) | 26.53 (6.89) |
| 1. Isolation | 10.74 (4.9) | 11.72 (5.7) | 10.55 (4.9) | 11.93 (5.9) | 11.59 (5.4) | 11.98 (5.7) | 10.89 (5.0) | 11.11 (5.4) |
| 1. Positive | 19.91 (5.7) | 19.65 (6.1) | 20.62 (5.8) | 19.34 (5.5) | 21.29 (5.8) | 20.09 (6.2) | 21.46 (5.7) | 19.84 (5.7) |
| 1. Negative | 18.4 (5.9) | 18.28 (5.8) | 17.29 (5.5) | 17.96 (6.2) | 18.21 (6.0) | 17.43 (6.0) | 17.45 (5.1) | 17.24 (5.2) |

**Table S3**

Comparisons of changes over time in mental health dependent on NDD diagnosis.

|  |  |  | *Depression* | | *Wellbeing* | | *Internalizing* | | *Externalizing* | |
| --- | --- | --- | --- | --- | --- | --- | --- | --- | --- | --- |
| **Effect** | **time** | **NDD Diagnosis** | **Estimate** | *p* | **Estimate** | *p* | **Estimate** | *p* | **Estimate** | *p* |
|  |  |  |  |  |  |  |  |  |  |  |
| **Intercept** |  |  | **57.979** | **<.0001** | 2.7748 | <.0001 | 6.0213 | <.0001 | 6.812 | <.0001 |
| **time** | **Post** |  | **-0.6523** | **0.6928** | 0.02083 | 0.7699 | 0.147 | 0.7502 | -0.2575 | 0.5827 |
| **time** | **Lockdown** |  | **1.8593** | **0.1854** | -0.115 | 0.0575 | 0.218 | 0.5817 | 0.4123 | 0.303 |
| **time** | **Pre** |  | **0** | **.** | 0 | . | 0 | . | 0 | . |
| **NDD Diagnosis** |  | **ADHD** | **5.1665** | **0.0131** | -0.2156 | 0.0175 | 1.6333 | 0.0051 | 3.015 | <.0001 |
| **NDD Diagnosis** |  | **ASD** | **1.9341** | **0.5241** | -0.2024 | 0.1202 | 0.5242 | 0.5448 | 1.1446 | 0.1835 |
| **NDD Diagnosis** |  | **SLD** | **1.4244** | **0.3616** | -0.05905 | 0.3803 | 0.8078 | 0.0661 | 0.767 | 0.0883 |
| **NDD Diagnosis** |  | **Other** | **0.9585** | **0.7895** | 0.2892 | 0.0605 | -0.6463 | 0.5194 | 0.6255 | 0.5386 |
| **NDD Diagnosis** |  | **No NDD** | **0** | **.** | 0 | . | 0 | . | 0 | . |
| **time* NDD Diagnosis** |  | **ADHD** | **-2.6222** | **0.4579** | 0.09416 | 0.5399 | -1.1464 | 0.2534 | -0.8108 | 0.4293 |
| **time* NDD Diagnosis** | **Lockdown** | **ASD** | **-0.06078** | **0.9894** | -0.05848 | 0.7677 | 0.3075 | 0.8131 | 0.351 | 0.7858 |
| **time* NDD Diagnosis** | **Lockdown** | **SLD** | **3.0231** | **0.2687** | -0.1441 | 0.2216 | -0.02449 | 0.9745 | 0.695 | 0.3735 |
| **time* NDD Diagnosis** | **Lockdown** | **Other** | **0.1148** | **0.9875** | -0.2291 | 0.4648 | 2.078 | 0.309 | -1.98 | 0.3391 |
| **time* NDD Diagnosis** | **Lockdown** | **No NDD** | **0** | **.** | 0 | . | 0 | . | 0 | . |
| **time* NDD Diagnosis** | **Post** | **ADHD** | **-2.6411** | **0.4329** | 0.3476 | 0.0153 | -1.1582 | 0.2122 | -1.6963 | 0.0741 |
| **time* NDD Diagnosis** | **Post** | **ASD** | **-0.7724** | **0.8665** | 0.1827 | 0.3537 | -0.3746 | 0.7725 | -1.8688 | 0.1514 |
| **time* NDD Diagnosis** | **Post** | **SLD** | **-1.0475** | **0.67** | 0.03658 | 0.7312 | 0.09962 | 0.8864 | -0.2652 | 0.7099 |
| **time* NDD Diagnosis** | **Post** | **Other** | **-7.3802** | **0.1791** | 0.05082 | 0.8291 | 0.8237 | 0.5914 | -0.7664 | 0.6222 |
| **time* NDD Diagnosis** | **Post** | **No NDD** | **0** | **.** | 0 | . | 0 | . | 0 | . |
| **time* NDD Diagnosis** | **Pre** | **ADHD** | **0** | **.** | 0 | . | 0 | . | 0 | . |
| **time* NDD Diagnosis** | **Pre** | **ASD** | **0** | **.** | 0 | . | 0 | . | 0 | . |
| **time* NDD Diagnosis** | **Pre** | **LD** | **0** | **.** | 0 | . | 0 | . | 0 | . |
| **time* NDD Diagnosis** | **Pre** | **Other** | **0** | **.** | 0 | . | 0 | . | 0 | . |
| **time* NDD Diagnosis** | **Pre** | **No NDD** | **0** | **.** | 0 | . | 0 | . | 0 | . |

**Appendix S3. *P* value calculation for Table S3**

| **Mental Health** | |  |  |
| --- | --- | --- | --- |
| **P-Values** | **Rank** | **Adjusted Threshold** | |
| 0.0153 | 1 | 0.007813 |  |
| 0.0741 | 2 |  |  |
| 0.1514 | 3 |  |  |
| 0.1791 | 4 |  |  |
| 0.2122 | 5 |  |  |
| 0.2216 | 6 |  |  |
| 0.2534 | 7 |  |  |
| 0.2687 | 8 |  |  |
| 0.309 | 9 |  |  |
| 0.3391 | 10 |  |  |
| 0.3537 | 11 |  |  |
| 0.3735 | 12 |  |  |
| 0.4293 | 13 |  |  |
| 0.4329 | 14 |  |  |
| 0.4579 | 15 |  |  |
| 0.4648 | 16 |  |  |
| 0.5399 | 17 |  |  |
| 0.5914 | 18 |  |  |
| 0.6222 | 19 |  |  |
| 0.67 | 20 |  |  |
| 0.7099 | 21 |  |  |
| 0.7312 | 22 |  |  |
| 0.7677 | 23 |  |  |
| 0.7725 | 24 |  |  |
| 0.7858 | 25 |  |  |
| 0.8131 | 26 |  |  |
| 0.8291 | 27 |  |  |
| 0.8665 | 28 |  |  |
| 0.8864 | 29 |  |  |
| 0.9745 | 30 |  |  |
| 0.9875 | 31 |  |  |
| 0.9894 | 32 |  |  |

**Table S4**

Mixed models for assessing change over time for each diagnosis.

|  |  | **Depression** | | | | | | | | | | | | | | | | | | | |
| --- | --- | --- | --- | --- | --- | --- | --- | --- | --- | --- | --- | --- | --- | --- | --- | --- | --- | --- | --- | --- | --- |
|  | Non-NDD | | | | |  | ASD | | |  | ADHD | | | | |  | SLD | | | |  |
|  | Estimate | | *p* | |  |  | Estimate | *p* |  |  | Estimate | *p* | |  | |  | Estimate | | *p* |  |  |
| **Time** |  | |  | |  |  |  |  |  |  |  |  | |  | |  |  | |  |  |  |
| Pre-COVID | (ref) | | - | |  |  | (ref) | - |  |  | (ref) | - | |  | |  | (ref) | | - |  |  |
| Schools Closed | 0.94 | | .342 | |  |  | 2.16 | .099 |  |  | -2.19 | .260 | |  | |  | 0.01 | | .996 |  |  |
| Schools Reopened | **2.12** | | **.009** | |  |  | 0.68 | .569 |  |  | -0.12 | .949 | |  | |  | 3.96 | | .054 |  |  |
|  |  | **Wellbeing** | | | | | | | | | | | | | | | | | | | |
|  | Non-NDD | | | |  |  | ASD | | |  | ADHD | | | | |  | SLD | | | |  |
|  | Estimate | | | *p* |  |  | Estimate | *p* |  |  | Estimate | | *p* | |  |  | Estimate | *p* | |  |  |
| **Time** |  | | |  |  |  |  |  |  |  |  | |  | |  |  |  |  | |  |  |
| Pre-COVID | (ref) | | | - |  |  | (ref) | - |  |  | (ref) | | - | |  |  | (ref) | - | |  |  |
| Schools Closed | -0.06 | | | .246 |  |  | -0.14 | .055 |  |  | 0.03 | | .673 | |  |  | -0.04 | .649 | |  |  |
| Schools Reopened | **-0.12** | | | **.005** |  |  | -0.07 | .249 |  |  | **0.23** | | **.003** | |  |  | 0.04 | .665 | |  |  |
|  |  | **Internalizing Symptoms** | | | | | | | | | | | | | | | | | | | |
|  | Non-NDD | | | | |  | ASD | | |  | ADHD | | | | |  | SLD | | | |  |
|  | Estimate | | *p* | |  |  | Estimate | *p* |  |  | Estimate | *p* | |  | |  | Estimate | | *p* |  |  |
| **Time** |  | |  | |  |  |  |  |  |  |  |  | |  | |  |  | |  |  |  |
| Pre-COVID | (ref) | | - | |  |  | (ref) | - |  |  | (ref) | - | |  | |  | (ref) | | - |  |  |
| Schools Closed | 0.31 | | .232 | |  |  | 0.29 | .444 |  |  | -0.80 | .213 | |  | |  | 0.44 | | .464 |  |  |
| Schools Reopened | 0.25 | | .236 | |  |  | 0.27 | .446 |  |  | -0.67 | .261 | |  | |  | 0.12 | | .841 |  |  |
|  |  | **Externalizing Symptoms** | | | | | | | | | | | | | | | | | | | |
|  | Non-NDD | | | |  |  | ASD | | |  | ADHD | | | | |  | SLD | | | |  |
|  | Estimate | | | *p* |  |  | Estimate | *p* |  |  | Estimate | | *p* | |  |  | Estimate | *p* | |  |  |
| **Time** |  | | |  |  |  |  |  |  |  |  | |  | |  |  |  |  | |  |  |
| Pre-COVID | (ref) | | | - |  |  | (ref) | - |  |  | (ref) | | - | |  |  | (ref) | - | |  |  |
| Schools Closed | 0.14 | | | .608 |  |  | 0.39 | .300 |  |  | -0.84 | | .110 | |  |  | 0.27 | .686 | |  |  |
| Schools Reopened | **0.66** | | | **.004** |  |  | 0.09 | .803 |  |  | **-1.15** | | **.019** | |  |  | -0.78 | .259 | |  |  |

*Note. (ref)* comparing magnitude of change at schools closed and schools reopened compared to pre-COVID.

**Appendix S4*. P* value calculation for Table S4**

| **P-Values** | **Rank** | **Adjusted Threshold** |
| --- | --- | --- |
| 0.003 | 1 | 0.007813 |
| 0.004 | 2 | 0.015625 |
| 0.005 | 3 | 0.023438 |
| 0.009 | 4 | 0.03125 |
| 0.019 | 5 | 0.039063 |
| 0.054 | 6 |  |
| 0.055 | 7 |  |
| 0.099 | 8 |  |
| 0.11 | 9 |  |
| 0.213 | 10 |  |
| 0.232 | 11 |  |
| 0.236 | 12 |  |
| 0.246 | 13 |  |
| 0.249 | 14 |  |
| 0.259 | 15 |  |
| 0.26 | 16 |  |
| 0.261 | 17 |  |
| 0.3 | 18 |  |
| 0.342 | 19 |  |
| 0.444 | 20 |  |
| 0.446 | 21 |  |
| 0.464 | 22 |  |
| 0.569 | 23 |  |
| 0.608 | 24 |  |
| 0.649 | 25 |  |
| 0.665 | 26 |  |
| 0.673 | 27 |  |
| 0.686 | 28 |  |
| 0.803 | 29 |  |
| 0.841 | 30 |  |
| 0.949 | 31 |  |
| 0.996 | 32 |  |

**Table S5**

Comparisons of changes over time in loneliness dependent on NDD diagnosis.

|  |  |  | *Friendship* | | *Isolation* | | *Positive* | | *Negative* | |
| --- | --- | --- | --- | --- | --- | --- | --- | --- | --- | --- |
| **Effect** | **time** | **NDD Diagnosis** | **Estimate** | *p* | **Estimate** | *p* | **Estimate** | *p* | **Estimate** | *p* |
|  |  |  |  |  |  |  |  |  |  |  |
| **Intercept** |  |  | 27.084 | <.0001 | 10.5462 | <.0001 | 20.6176 | <.0001 | 17.2941 | <.0001 |
| **time** | **Post** |  | -0.2605 | 0.7348 | 1.042 | 0.1086 | 0.6765 | 0.3236 | 0.9118 | 0.1758 |
| **time** | **Lockdown** |  | 0.07573 | 0.9078 | 0.3414 | 0.5361 | 0.8471 | 0.1454 | 0.1529 | 0.7889 |
| **time** | **Pre** |  | 0 | . | 0 | . | 0 | . | 0 | . |
| **NDD Diagnosis** |  | **ADHD** | -3.0295 | 0.0019 | 2.1447 | 0.0091 | -1.7631 | 0.042 | 1.9059 | 0.0253 |
| **NDD Diagnosis** |  | **ASD** | -2.0406 | 0.1506 | 1.5407 | 0.1984 | -0.7046 | 0.5773 | 0.9668 | 0.4364 |
| **NDD Diagnosis** |  | **SLD** | -0.06723 | 0.9266 | 1.5042 | 0.0147 | -1.105 | 0.0894 | 0.1176 | 0.8538 |
| **NDD Diagnosis** |  | **Other** | 1.166 | 0.4872 | -0.1087 | 0.9388 | -1.2426 | 0.406 | -0.2941 | 0.8413 |
| **NDD Diagnosis** |  | **No NDD** | 0 | . | 0 | . | 0 | . | 0 | . |
| **time* NDD Diagnosis** |  | **ADHD** | 0.706 | 0.6662 | -0.5767 | 0.6763 | -1.9373 | 0.1842 | -0.9243 | 0.5189 |
| **time* NDD Diagnosis** | **Lockdown** | **ASD** | 0.917 | 0.6669 | -0.979 | 0.5861 | 0.4105 | 0.8288 | -1.9226 | 0.3027 |
| **time* NDD Diagnosis** | **Lockdown** | **SLD** | -0.7719 | 0.5425 | -1.3112 | 0.2204 | 1.0765 | 0.3405 | -0.9798 | 0.3772 |
| **time* NDD Diagnosis** | **Lockdown** | **Other** | 0.4105 | 0.9044 | -1.6795 | 0.5603 | 3.1485 | 0.3012 | -3.7118 | 0.2148 |
| **time* NDD Diagnosis** | **Lockdown** | **No NDD** | 0 | . | 0 | . | 0 | . | 0 | . |
| **time* NDD Diagnosis** | **Post** | **ADHD** | 2.1197 | 0.1685 | -1.5323 | 0.2382 | -1.9238 | 0.1607 | 0.6193 | 0.6456 |
| **time* NDD Diagnosis** | **Post** | **ASD** | -0.1192 | 0.9557 | -1.9839 | 0.2737 | -1.149 | 0.548 | -2.0249 | 0.2813 |
| **time* NDD Diagnosis** | **Post** | **SLD** | -0.4343 | 0.7051 | -0.9361 | 0.3338 | 0.2226 | 0.8275 | -1.1596 | 0.2482 |
| **time* NDD Diagnosis** | **Post** | **Other** | -1.7424 | 0.4972 | -0.3622 | 0.8672 | 1.6946 | 0.4586 | 0.5971 | 0.7904 |
| **time* NDD Diagnosis** | **Post** | **No NDD** | 0 | . | 0 | . | 0 | . | 0 | . |
| **time* NDD Diagnosis** | **Pre** | **ADHD** | 0 | . | 0 | . | 0 | . | 0 | . |
| **time* NDD Diagnosis** | **Pre** | **ASD** | 0 | . | 0 | . | 0 | . | 0 | . |
| **time* NDD Diagnosis** | **Pre** | **LD** | 0 | . | 0 | . | 0 | . | 0 | . |
| **time* NDD Diagnosis** | **Pre** | **Other** | 0 | . | 0 | . | 0 | . | 0 | . |
| **time* NDD Diagnosis** | **Pre** | **No NDD** | 0 | . | 0 | . | 0 | . | 0 | . |

**Appendix S5. *P* value calculation for Table S5**

| **Loneliness** |  |
| --- | --- |
| **P-Values** | **Rank** |
| 0.1607 | 1 |
| 0.1685 | 2 |
| 0.1842 | 3 |
| 0.2148 | 4 |
| 0.2204 | 5 |
| 0.2382 | 6 |
| 0.2482 | 7 |
| 0.2737 | 8 |
| 0.2813 | 9 |
| 0.3012 | 10 |
| 0.3027 | 11 |
| 0.3338 | 12 |
| 0.3405 | 13 |
| 0.3772 | 14 |
| 0.4586 | 15 |
| 0.4972 | 16 |
| 0.5189 | 17 |
| 0.5425 | 18 |
| 0.548 | 19 |
| 0.5603 | 20 |
| 0.5861 | 21 |
| 0.6456 | 22 |
| 0.6669 | 23 |
| 0.6763 | 24 |
| 0.7051 | 25 |
| 0.7904 | 26 |
| 0.8275 | 27 |
| 0.8288 | 28 |
| 0.8672 | 29 |
| 0.9044 | 30 |
| 0.9557 | 31 |
|  | 32 |
